# Development of a (Poly)phenol Metabolic Signature for Assessing (Poly)phenol-Rich Dietary Patterns

**DOI:** 10.1021/acs.jafc.4c00959

**Published:** 2024-06-03

**Authors:** Yong Li, Yifan Xu, Melanie Le Sayec, Xinyu Yan, Tim D. Spector, Claire J. Steves, Jordana T. Bell, Kerrin S. Small, Cristina Menni, Rachel Gibson, Ana Rodriguez-Mateos

**Affiliations:** † Department of Nutritional Sciences, School of Life Course and Population Sciences, Faculty of Life Sciences and Medicine, King’s College London, London SE1 9NH, U.K.; ‡ Department of Twin Research & Genetic Epidemiology, School of Life Course and Population Sciences, Faculty of Life Sciences and Medicine, King’s College London, London SE1 7EH, U.K.

**Keywords:** (poly)phenol-rich dietary score, metabolic signature, food frequency questionnaire, 7-day food diary

## Abstract

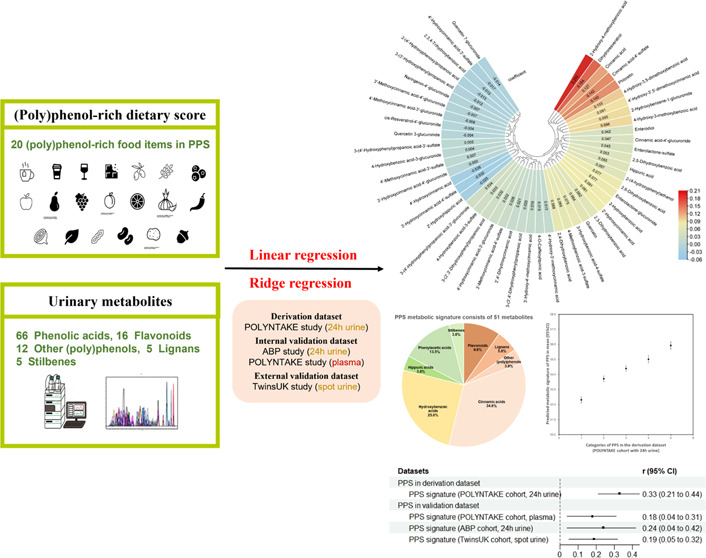

The objective assessment of habitual (poly)phenol-rich diets in nutritional epidemiology studies remains challenging. This study developed and evaluated the metabolic signature of a (poly)phenol-rich dietary score (PPS) using a targeted metabolomics method comprising 105 representative (poly)phenol metabolites, analyzed in 24 h of urine samples collected from healthy volunteers. The metabolites that were significantly associated with PPS after adjusting for energy intake were selected to establish a metabolic signature using a combination of linear regression followed by ridge regression to estimate penalized weights for each metabolite. A metabolic signature comprising 51 metabolites was significantly associated with adherence to PPS in 24 h urine samples, as well as with (poly)phenol intake estimated from food frequency questionnaires and diaries. Internal and external data sets were used for validation, and plasma, spot urine, and 24 h urine samples were compared. The metabolic signature proposed here has the potential to accurately reflect adherence to (poly)phenol-rich diets, and may be used as an objective tool for the assessment of (poly)phenol intake.

## Introduction

(Poly)phenols, as a large family of plant secondary metabolites, are distributed broadly in the plant kingdom and are present in almost all plant foods and beverages.^1^ Growing evidence indicates that (poly)phenols have a significant role in reducing the risk of multiple chronic diseases, such as cardiovascular disease, type-2 diabetes, and age-related cognitive decline.^2−4^ However, estimating (poly)phenol consumption accurately remains challenging, and this can be an important confounding factor when establishing health benefits, particularly in epidemiological studies.^5^ To overcome such issues, the use of dietary biomarkers as objective measurements of dietary intake has been proposed as an alternative to dietary assessment tools. Biomarkers have been used to estimate the consumption of specific foods, nutrients, or bioactive compounds, or to develop calibrated equations to correct intake estimated from food frequency questionnaires (FFQ) or food records.^6^ However, very few validated biomarkers exist, and it is very challenging to find suitable biomarkers for each individual food/component within the diet.

The field of metabolomics has emerged as a powerful tool in nutritional research in the last few decades to identify a group of biomarkers linked to specific dietary patterns. A metabolomic profiling approach can be employed to construct a metabolic signature for characterizing a certain diet and reflecting adherence to a specific dietary pattern.^7,8^ Although several studies have proposed signatures for dietary patterns, including the Mediterranean Diet Score (MDS)^7,9^ and the Healthy Eating Index (HEI)-2020,^10^ metabolic signature data of habitual diets in this field remains limited. Currently, the relationship between (poly)phenol metabolite levels and the consumption of (poly)phenol-rich diets is poorly understood, and little research has been conducted so far on whether (poly)phenol metabolites in biofluids can predict the intake of (poly)phenol-rich foods.^11^ Metabolomics studies with a combination of metabolites from a comprehensive profile of all (poly)phenol classes may improve the prediction of dietary (poly)phenol intake.

We have recently developed a (poly)phenol-rich diet score (PPS) to reflect adherence to a (poly)phenol-rich diet in free-living individuals.^12^ In this work, we aim to establish a metabolic signature of a (poly)phenol-rich diet using the PPS score and a comprehensive high-throughput targeted metabolomics method for the quantification of a large number of (poly)phenol metabolites in urine and plasma^13^ in healthy free-living individuals.

## Materials and Methods

### Study Population

The POLYNTAKE cohort comprises healthy participants who participated in nine dietary intervention studies conducted at King’s College London from 2017 to 2021 (Ethics number, RESCM-17/18-5283; HR-15/16-3739; HR-17/18-5338; HR-18/19-9091; HR-18/19-8999; HR-17/18-5703; RESCM-18/19-9036; HR-17/18-5353; HR-19/20-14771; Trial registration number, NCT03434574; NCT03041961; NCT03592966; NCT04084457; NCT04179136; NCT03553225; NCT03995602; NCT03573414; NCT04276974). These studies were conducted in accordance with the Declaration of Helsinki, with all participants providing informed written consent and agreeing to the use of data in future studies. This cross-sectional secondary analysis included participants who completed food frequency questionnaires and provided blood and/or urine samples that were analyzed for the quantification of (poly)phenol metabolites using liquid chromatography–mass spectrometry (LC–MS). As previously described,^14^ to eliminate outliers in dietary intake, we excluded (i) participants with more than ten missing food items from the FFQ; (ii) females with daily caloric intake below 500 kcal or higher than 3500 kcal; (iii) males with daily caloric intake below 800 kcal or higher than 4000 kcal;^15^ and (iv) participants with an energy intake to basal metabolic rate ratio outside the mean ±2 standard deviations (SD) of the population, as estimated by the Harris–Benedict equation according to the Goldberg method.^16^ A total of 229 participants were included in the analysis, with 204 participants providing plasma samples and 229 providing 24 h urine samples.

A total of 229 participants from the POLYNTAKE study with 24 h urine samples were used to develop the metabolic signature (“the derivation set”). The 24 h urine samples from participants from the subcohort Aronia Berry Consumption on Blood Pressure study (ABP), which is one of the studies included in the POLYNTAKE study, were used as an internal validation set, consisting of 95 participants after exclusion. Plasma samples from the POLYNTAKE cohort, including 204 subjects, were also used as an internal validation set. Additionally, 198 free-living individuals from TwinsUK cohort, with spot urine samples, were used as an external validation set with the ethical approval from the NHS Research Ethics Committee at the Department of Twin Research and Genetic Epidemiology, King’s College London (the Healthy Aging Twin Study (H.A.T.S) 07/H0802/84) and the NRES Committee London-Westminster (Flora Twin Study reference 12/LO/0227).^17^ Detailed information on the above studies can be found in Supporting Information, Table S1.

### Dietary Assessment Tools

A self-administered European Prospective Investigation into Cancer (EPIC)-Norfolk FFQ was completed by the participants at the baseline visit to capture their diet before any intervention. This FFQ, designed and validated for estimating key nutrients and energy intake in the past year among UK adults, was employed in the EPIC-Norfolk study.^18,19^ The questionnaire records the dietary intake of 130 food items with 9 frequency options, ranging from “never or less than once a month” to “more than 6 times per day”. Additionally, details regarding the types of milk, cereals, cooking fat, and the amount of visible fat consumed in meals were explored in a separate section of the questionnaire. The FFQ EPIC Tool for Analysis (FETA) software was used to calculate the energy intake and daily food consumption. FETA includes composition data for 290 foods from the UK food database McCance and Widdowson’s “The Composition of Foods” (fifth edition)^20^ and its associated supplements. An average portion size was assigned to each questionnaire item. After the frequency of intake was multiplied by the portion size, the average daily food consumption (g/d) for each of the 130 FFQ items was obtained.

Participants from the POLYNTAKE cohort were provided with a 7-day food diary (7DD) to record their foods and drinks in a consecutive 7-day period, 1 week before their baseline appointment.^21^ The booklet was divided into six sections: before breakfast, breakfast, midmorning, lunch, tea, evening meal, and later evening. Standard photographs of portion sizes, along with instructions and examples, were provided on the initial pages of the 7DDs to help subjects record their diets in as much detail as possible.

### Estimation of Dietary (Poly)phenol Intake

(Poly)phenol intake was calculated from food intake (g/d) estimated from both FFQs and 7DDs coupled with the corresponding content in foods (mg/100g, aglycones equivalent) derived from a (poly)phenol database (PPDB) developed at the Department of Nutritional Sciences of King’s College London, which has been described in detail previously.^21^ The PPDB integrated (poly)phenol content data of 1260 raw and processed food items or dishes, which were obtained from multiple sources such as the Phenol-Explorer database,^22^ USDA databases,^23−25^ and published data. The (poly)phenol content for composition dishes was calculated based on recipes from McCane and Widdowson’s (sixth edition) Supporting Information ^20^ and information on retailer websites. Food items such as animal products with little or no (poly)phenol content were removed from the calculation. The total subclass, class, and total (poly)phenol intake were calculated by summarizing the intake of all of the compounds within the group.

### (Poly)phenol-Rich Dietary Score Assessment with FFQs

We calculated the (poly)phenol-rich diet score (PPS) to estimate adherence to a (poly)phenol-rich diet.^12^ The PPS was designed based on the relative intake levels of 20 (poly)phenol-rich food items from the EPIC-Norfolk FFQ, including tea, coffee, red wine, wholegrains, breakfast cereals, chocolate and cocoa products, berries, apples and apple juice, pears, grapes, plums, citrus fruits and citrus juice, potatoes and carrots, onions, peppers, garlic, green vegetables, pulses, soybeans and related products, nuts, and olive oil.

Participants were scored by quintiles of their intake of each food group (g/d) in the study population. The subjects in the highest quintile among the study population scored 5, and the subjects in the lowest quintile scored 1. The PPS was calculated as the total scores of all 20 food groups ranging from 20 to 100. All food groups were assigned the same level of weight.^12^

### Sample Collection and Metabolite Analysis

All plasma and urine (poly)phenol metabolite data presented here were analyzed using the same methodology. In the POLYNTAKE cohort, 24 h urine (*n* = 229) and fasting plasma samples (*n* = 204) were collected to analyze the metabolite levels. The collection, storage, processing, and UHPLC-MS analysis with a triple-quadruple mass spectrometer (SHIMADZU 8060, Shimadzu, Kyoto, Japan) of the 24 h urine and fasting plasma samples have been described in detail previously.^12,13^ A total of 105 urinary and 114 plasma metabolites were identified and quantified with authentic chemical standards. The spot urine samples in the TwinsUK cohort (*n* = 198) were processed and analyzed using the same method and device mentioned above.^12,17^ The urinary creatinine levels were analyzed with the Jaffe method by Affinity Biomarker Laboratories (London, U.K.), and the metabolite levels (nM) were adjusted by creatinine (mg/L) into mmol/g creatinine.^17^

### Statistical Analysis

We used R software (version 4.1.2) for statistical analysis.^26^ Metabolite levels were log-transformed and adjusted for batch effects using the ComBat method (from the sva package in R).^27,28^ We adjusted for energy intake estimated from FFQs in the linear regression model to explore the association between PPS and metabolites (lm.beta package in R). All analyses were adjusted for multiple testing (Benjamini and Hochberg false discovery rate (FDR) < 0.05, FDR suggestive significant 0.05–0.10).^29^ Metabolites with significant associations with PPS were selected as the next step.

Metabolic signatures of PPS were constructed to represent adherence to (poly)phenol-rich diet based on selected individual significant metabolites, with penalized weights estimated through ridge regression^30^ in the derivation data set (24 h urine samples from the POLYNTAKE study). To investigate whether it was possible to replicate the metabolic signatures of PPS in other studies, 3 validation data sets were used: the ABP study (internal subcohort of the POLYNTAKE study with 24 h urine samples), the plasma samples from the POLYNTAKE study, and the external TwinsUK data set with spot urine samples (Supporting Information, Table S1).

**Figure 1:**
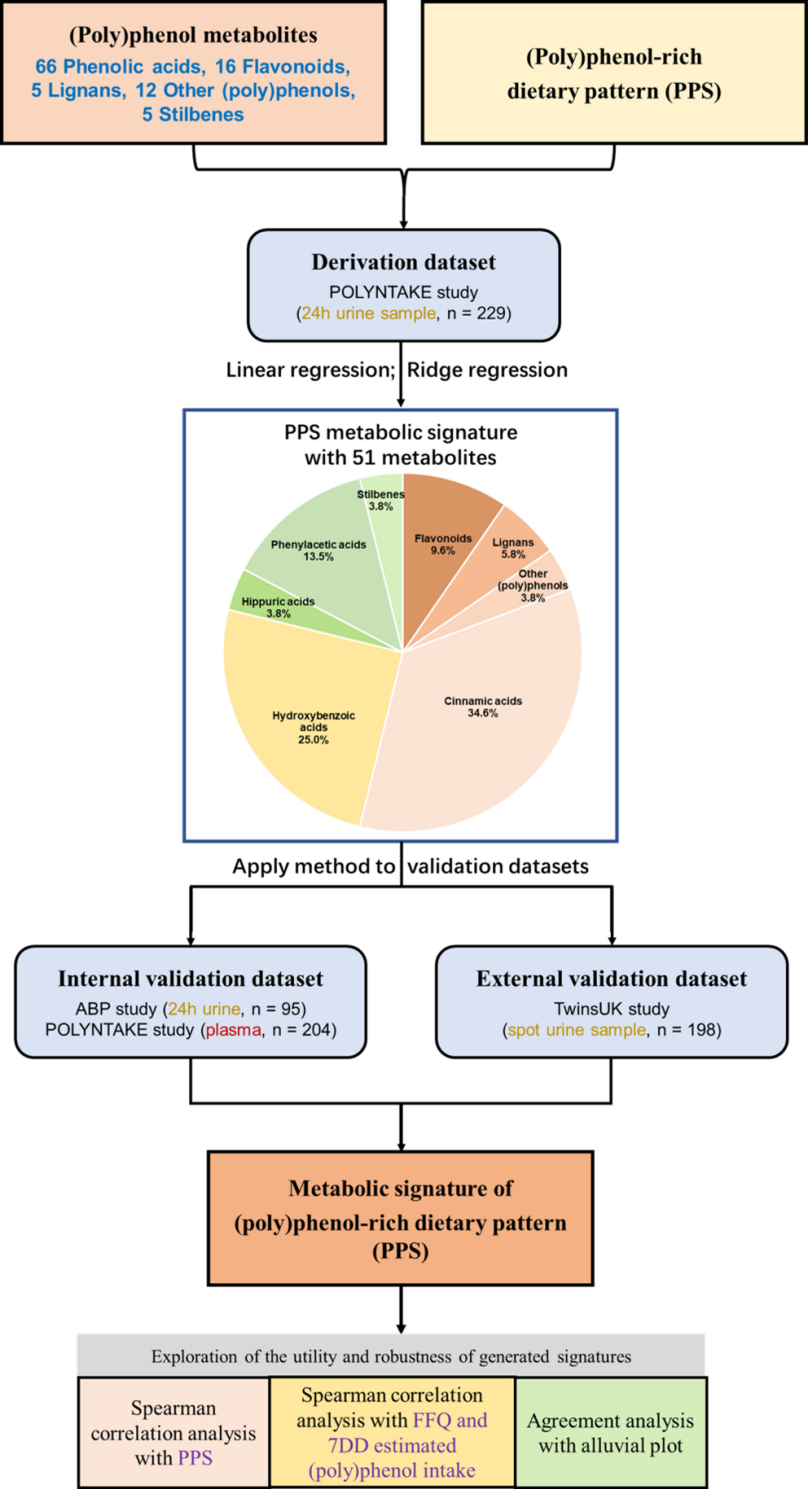
Flowchart of the analysis approach (generation and validation) to develop the metabolic signature to estimate adherence to (poly)phenol-rich dietary patterns. PPS, (poly)phenol-rich dietary score; FFQ, food frequency questionnaire; and 7DD, 7-day food diary.

To explore the utility and robustness of the metabolite signatures, the correlation between PPS and its metabolic signature was tested by Spearman correlation analyses in both derivation and validation data sets. Spearman correlations were also employed to assess the association between the PPS signature and FFQ and 7DD estimated (poly)phenol intake in each data set. To further examine the agreement between PPS and metabolite signatures, alluvial plots were constructed to illustrate the cross-classification results between quartiles. A detailed flowchart of the analysis is exhibited in Figure 1.

## Results

### Characteristics of Study Population

The characteristics of the participants from the POLYNTAKE cohort are listed in Table 1. The average age of the subjects was 52.3 (SD 17.6) years. The majority of subjects were from the white ethnic group (76.0%), and their average energy intake was 1586.9 kcal/d (SD 465.6). The 24 h urine samples were collected from 99 males and 130 females, whereas plasma samples were collected from 89 males and 115 females. The average PPS was 54.6 (SD 11.5), and total (poly)phenol intake estimated from FFQs and 7DDs were 1635.6 (899.1) mg/day, and 1707.0 (963.8) mg/day, respectively.

**Table 1:** Characteristics of the Study Population of the POLYNTAKE Cohort

characteristics	men mean (SD)/*n* (%)	women mean (SD)/*n* (%)	total mean (SD)/*n* (%)
age (years)	52.5 (16.9)	52.1 (18.2)	52.3 (17.6)
ethnicity			
white	72 (72.7)	102 (78.5)	174 (76.0)
black	7 (7.1)	6 (4.6)	13 (5.7)
asian	13 (13.1)	21 (16.2)	34 (14.8)
mixed	7 (7.1)	1 (0.7)	8 (3.5)
energy intake (kcal/day)	1724.4 (486.0)	1474.5 (417.6)	1586.9 (465.6)
sample (*n*)			
24 h urine	99 (43.2)	130 (56.8)	229
plasma^a^	89 (43.6)	115 (56.4)	204
PPS and estimated total (poly)phenol			
PPS	52.5 (11.0)	56.2 (11.6)	54.6 (11.5)
total (poly)phenol estimated from FFQs (mg/day)	1620.2 (928.3)	1647.3 (879.8)	1635.6 (899.1)
total (poly)phenol estimated from 7DDs (mg/day)	1652.0 (994.1)	1750.1 (942.8)	1707.0 (963.8)

a The number of missing plasma samples is 25. PPS, (poly)phenol-rich dietary score; FFQ, food frequency questionnaire; 7DD, 7-day food diary; and SD, standard deviation.

The characteristics of the participants in the ABP and TwinsUK cohorts are shown in Supporting Information, Table S2. The average age of subjects was 56.4 (SD 8.9) years in the ABP cohort and 62.0 (SD 9.9) years in the TwinsUK cohort. Most of the subjects were from the white ethnic group (ABP: 80.0% and TwinsUK: 99.0%), and their average energy intake was 1681.3 kcal/day (SD 490.7) and 1782.5 kcal/day (SD 554.3) in ABP and TwinsUK study, respectively. The 24 h urine samples in the ABP cohort were collected from 46 males and 49 females, and the spot urine samples (*n* = 198) in the TwinsUK cohort were all collected from females.

### Correlation between PPS, FFQ, and 7DD Estimated (Poly)phenol Intake

The correlation between PPS, FFQ, and 7DD estimated (poly)phenol intake is shown in Supporting Information, Figure S1. The correlation coefficients ranged from 0.10 to 0.86, among which the strongest correlation was observed between phenolic acids estimated from FFQs and total (poly)phenol estimated from FFQs (*r* = 0.86), and the weakest correlation was found between phenolic acids estimated from FFQs and other (poly)phenols estimated from FFQs (*r* = 0.10).

### Associations between PPS, FFQ and 7DD Estimated Total (Poly)phenol Intake and Metabolites

**Figure 2:**
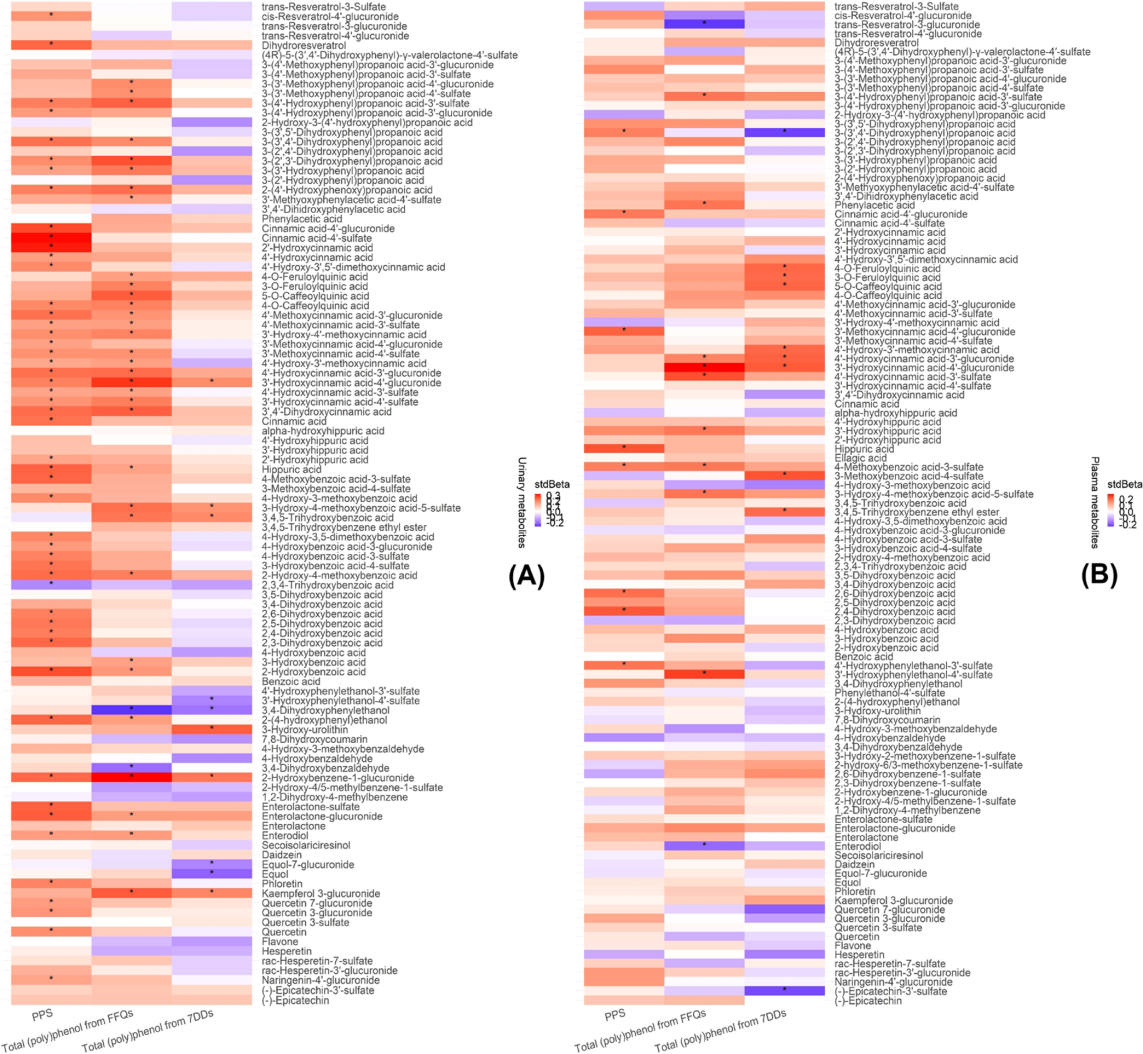
Association between PPS, FFQ, and 7DD estimated total (poly)phenol intake and (A) urinary and (B) plasma metabolites. A heatmap was plotted according to the standardized regression coefficients (stdBeta). The color scale indicates the effect (stdBeta) of each metabolite on the PPS, FFQ, and 7DD estimated total (poly)phenol intake. Red and blue indicate positive and negative effects, and the color intensity represents the degree of effect. Asterisks indicate significance (*: FDR-adjusted *p* < 0.05). PPS, (poly)phenol-rich dietary score; FFQs, food frequency questionnaires; and 7DDs, 7-day food diaries. The associations were adjusted for energy intake.

The association between 105 urinary metabolites and PPS, FFQ and 7DD estimated total (poly)phenol intake is shown in Figure 2A. The significant standardized regression coefficients (and 95% CI) between urinary metabolites and PPS were all positive (*n* = 51), ranging from 0.14 (0.004, 0.29) for 4′-hydroxy-3′-methoxycinnamic acid to 0.32 (0.19, 0.56) for cinnamic acid-4′-sulfate, except for stdBeta for 2,3,4-trihydroxybenzoic acid, which was negative [−0.16 (−0.30, −0.02), FDR-adjusted *p* < 0.05].

The total (poly)phenol intake estimated from the FFQ was positively associated with 33 urinary metabolites with significant standardized regression coefficients (and 95% CI) ranging from 0.15 (0.01, 0.29) for 4′-hydroxycinnamic acid to 0.33 (0.19, 0.46) for 2-hydroxybenzene-1-glucuronide, except for 3,4-dihydroxyphenylethanol [−0.28 (−0.41, −0.14)] and 3,4-dihydroxybenzaldehyde [−0.20 (−0.34, −0.07)], which were negatively associated (FDR-adjusted *p* < 0.05). Phenolic acids contributed the most to the positive associations with PPS and total (poly)phenol from the FFQ, including 39 metabolites associated with PPS and 30 associated with total (poly)phenol from the FFQ (all FDR-adjusted *p* < 0.05). The total (poly)phenol intake estimated from 7DD was associated with 10 metabolites, including 3 flavonoids, 3 phenolic acids, 2 tyrosols, 1 benzene diols and triol, and hydroxycoumarins (all FDR-adjusted *p* < 0.05).

The association between the 111 plasma metabolites and PPS, FFQ, and 7DD estimated total (poly)phenol intake is presented in Figure 2B. The significant associations between FFQ and 7DD estimated total (poly)phenol intake and plasma metabolites were mainly positive, except for total (poly)phenol estimated from FFQ and *trans*-resveratrol-3-glucuronide [−0.24 (−0.34, −0.09)], enterodiol [−0.18 (−0.32, −0.03)], and total (poly)phenol estimated from 7DD and 3-(3′,4′-dihydroxyphenyl)propanoic acid [−0.22 (−0.39, −0.06)], (−)-epicatechin-3′-sulfate [−0.21 (−0.39, −0.04)] (all FDR-adjusted *p* < 0.05). The significant standardized regression coefficients (and 95% CI) between plasma metabolites and PPS were all positive (*n* = 8), ranging from 0.18 (0.03, 0.32) for 3-(3′,4′-dihydroxyphenyl)propanoic acid to 0.23 (0.08, 0.38) for hippuric acid (all FDR-adjusted *p* < 0.05). The total (poly)phenol intake estimated from FFQ was positively associated with nine plasma metabolites, including eight phenolic acids and one tyrosol, with significant standardized regression coefficients (and 95% CI) ranging from 0.17 (0.03, 0.32) for 4′-hydroxycinnamic acid-3′-glucuronide to 0.28 (0.14, 0.42) for 3′-hydroxycinnamic acid-4′-glucuronide (FDR-adjusted *p* < 0.05). The total (poly)phenol intake estimated from 7DD was positively associated with eight plasma metabolites, including six hydroxycinnamic acids and two hydroxybenzoic acids, with significant standardized regression coefficients (and 95% CI) ranging from 0.20 (0.03, 0.38) for 3-*O*-feruloylquinic acid to 0.23 (0.06, 0.40) for 3-methoxybenzoic acid-4-sulfate (FDR-adjusted *p* < 0.05).

The association between intake of classes of (poly)phenols estimated from FFQs and 7DDs and urinary metabolites was further explored, as shown in Supporting Information, Figure S2A,B. Phenolic acid intake was associated with the highest number of metabolites among all classes, with a total of 36 and 14 individual metabolites associated with phenolic acid intake estimated from FFQs and 7DDs, respectively (all FDR-adjusted *p* < 0.05). The significant standardized regression coefficients (and 95% CI) between urinary metabolites and phenolic acids from both FFQs and 7DDs were all positive (*n* = 49), ranging from 0.16 (0.03, 0.30) for 3-hydroxybenzoic acid to 0.45 (0.34, 0.57) for 2-hydroxybenzene-1-glucuronide with phenolic acids from FFQs, except for stdBeta for 3,4-dihydroxyphenylethanol, which was negative [−0.23 (−0.37, −0.10), FDR-adjusted *p* < 0.05].

As for other classes, lignans, flavonoids, other (poly)phenols, and stilbene intake estimated from FFQs were significantly associated with 13, 7, 6, and 9 urinary metabolites, respectively, with only 5 negative associations in the class of flavonoids and stilbenes, FDR-adjusted (*p* < 0.05). As for other classes of (poly)phenols estimated from 7DDs, lignans, flavonoids, other (poly)phenols, and stilbenes were significantly associated with 7, 10, 3, and 1 urinary metabolite, respectively. Negative associations were found for the class of flavonoids and other (poly)phenols to −0.29 (−0.44, −0.13) for *trans*-resveratrol-3-sulfate and other (poly)phenols to −0.21 (−0.36, −0.04) for 4′-hydroxycinnamic acid-3′-sulfate and flavonoids, FDR-adjusted (*p* < 0.05).

### Development and Validation of a Metabolic Signature for Estimating Adherence to a (Poly)phenol-Rich Diet

**Figure 3:**
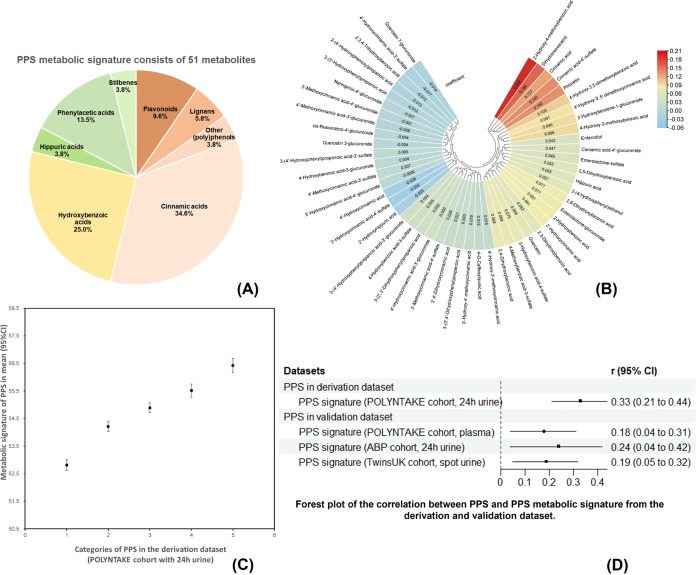
Selected metabolites included in the PPS metabolic signature and the correlations between PPS and PPS metabolic signatures from the derivation and validation data set. (A) Percentage of the metabolites in the PPS metabolic signature from the derivation data set. The metabolic signature of PPS was derived based on selected metabolites that were significantly associated with PPS. (B) The coefficient of selected metabolites from the PPS in the derivation data set. Color intensity represents the degree of coefficient of each metabolite. (C) Mean and 95% CI of the derived metabolic signature (51 metabolites) by quintiles of PPS. (D) Forest plot of the correlation between PPS and PPS metabolic signatures from derivation and validation data sets. PPS, (poly)phenol-rich dietary score.

The 24 h urine sample was chosen to establish the metabolic signature, as it had the highest number of metabolites associated with PPS and had stronger correlations than plasma. Figure 3A shows the percentages of the classes and subclasses of metabolites included in the PPS metabolic signature. Hydroxycinnamic acids and hydroxybenzoic acids, among the subclasses of phenolic acids, accounted for 34.6 and 25.0% in the composition of PPS signature. Figure 3B shows the coefficients of the classes and subclasses for each selected metabolite in the metabolic signature. The scatter plot in Figure 3C identifies a positive visual relationship between the PPS in quintiles and the metabolic signature. In Figure 3D, a Spearman correlation was implemented to assess the statistical correlation between PPS and its metabolic signature in the derivation data set of the POLYNTAKE cohort using 24 h urine samples. In addition, Spearman correlations were also tested in the validation data sets with three sample types across three cohorts (the POLYNTAKE cohort with plasma samples, the ABP cohort with 24 h urine samples, and the TwinsUK cohort with spot urine samples). The strongest correlation was found between PPS and its metabolic signature in the derivation data set [0.33 (0.21, 0.44)], followed by the correlation in the ABP cohort [0.24 (0.04, 0.42)], TwinsUK cohort [0.19 (0.05, 0.32)], and POLYNTAKE cohort using plasma [0.18 (0.04, 0.31)] (FDR-adjusted *p* < 0.05). In the derivation cohort, the correlation between the PPS and metabolic signature was higher than that of the individual 51 significant metabolites (Supporting Information, Table S3).

### Agreements between PPS Derived from FFQs and Metabolite Signatures

**Figure 4:**
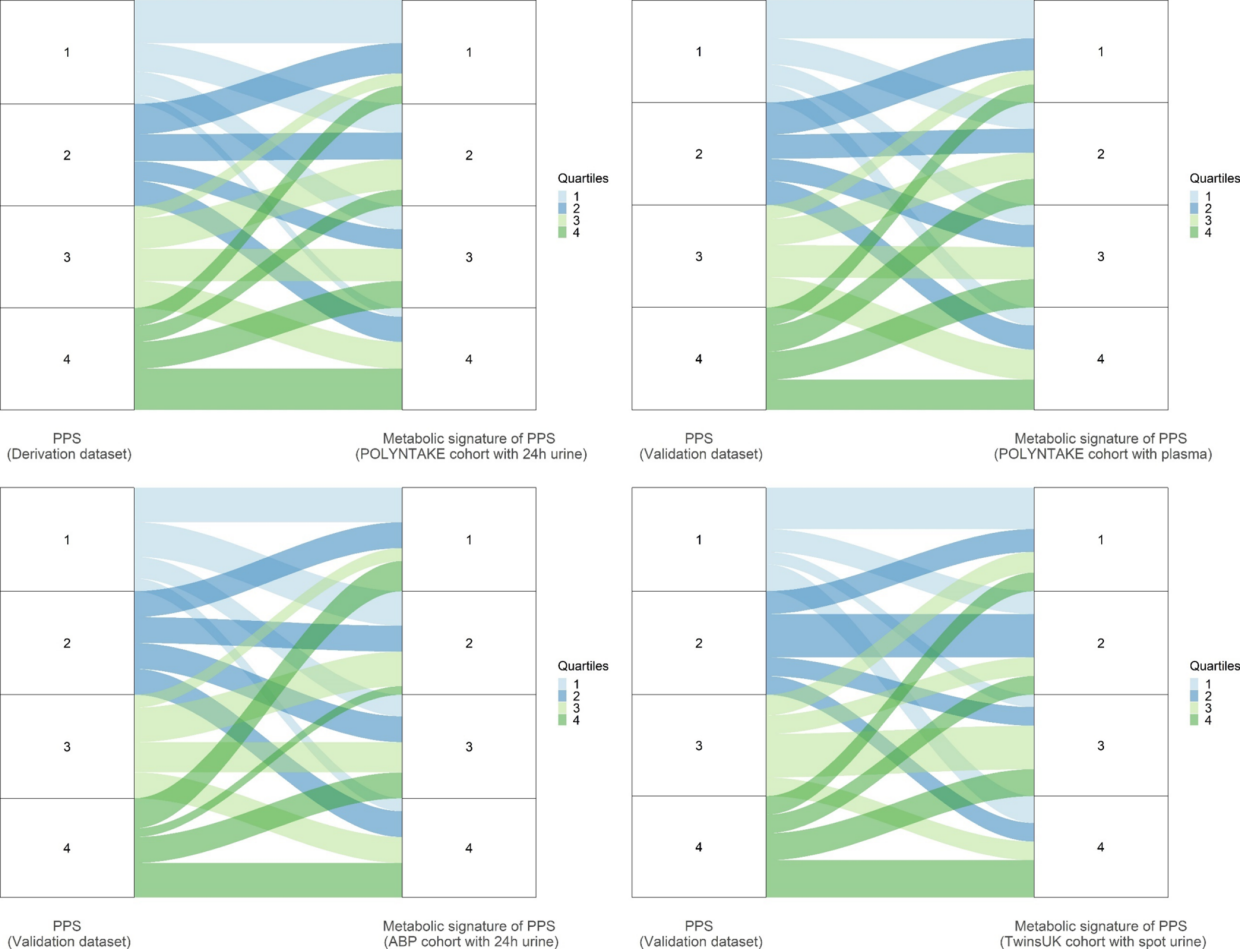
Agreements between (poly)phenol and the metabolic signature in ranking participants into quartiles. PPS, (poly)phenol-rich dietary score.

The agreement between PPS derived from FFQs and PPS metabolic signatures when ranking participants into quartiles in the derivation and validation data sets is shown in Figure 4. The two methods were comparable in differentiating participants into high and low adherence to the PPS dietary pattern in the derivation data set POLYNTAKE cohort (24 h urine), the validation data set POLYNTAKE cohort (plasma), ABP cohort (24 h urine), and TwinsUK cohort (spot urine) with 74.7, 70.6, 72.6, and 71.7% of participants ranked into the same quartile or adjacent quartile and 6.6, 8.8, 10.5, and 11.1% ranked into the opposite quartile (the first and fourth quartiles), respectively.

### (Poly)phenol Metabolic Signatures and FFQ and 7DD Estimated (Poly)phenol Intake

**Figure 5:**
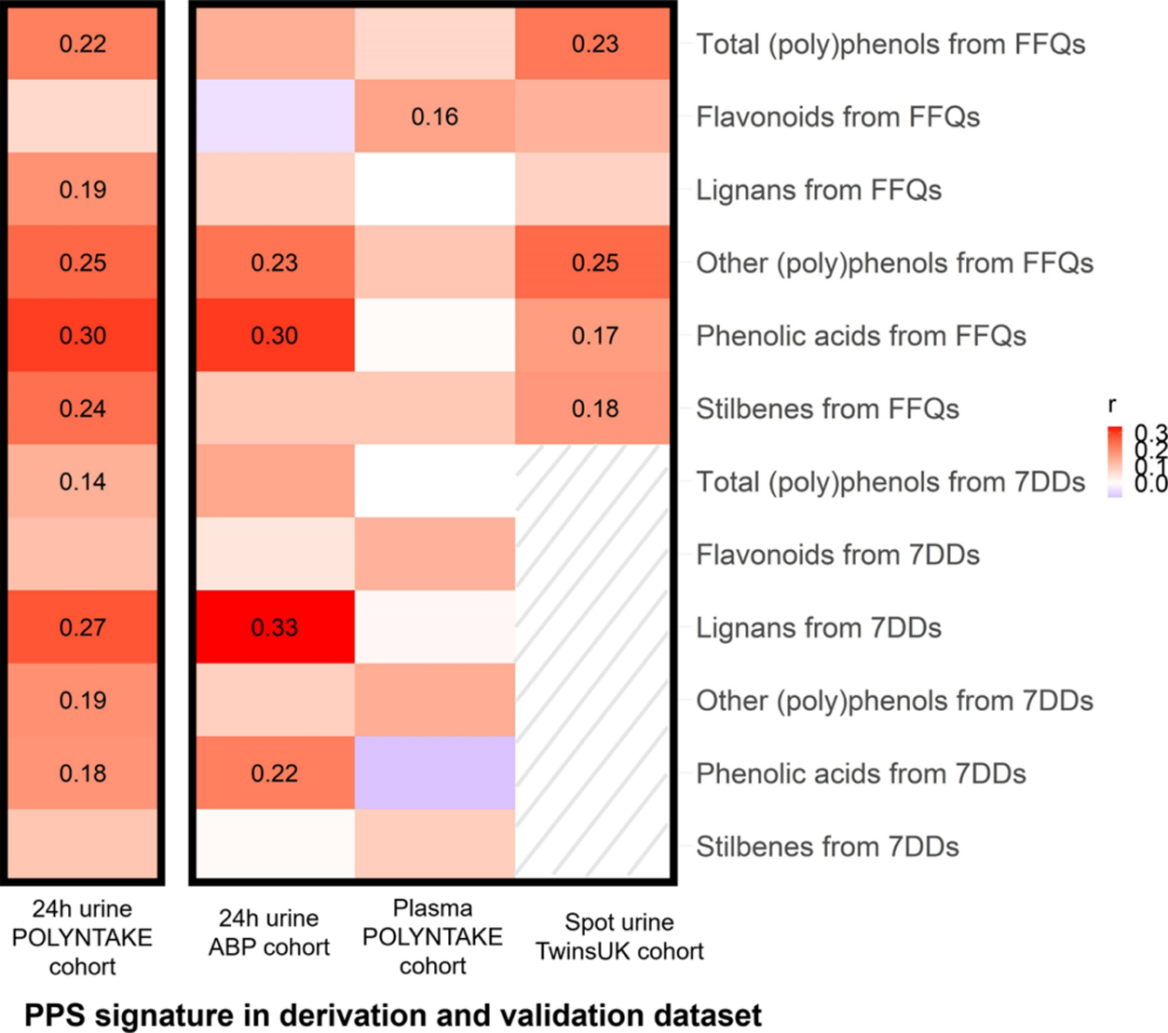
Correlation between PPS metabolic signature and FFQ and 7DD estimated (poly)phenol intake in derivation and validation data sets. Red and blue indicate positive and negative effects, respectively, and the color intensity represents the degree of effect. 7DDs were not collected in the TwinsUK study, so no correlations are shown between the (poly)phenol signature and 7DD estimated (poly)phenol intake in the TwinsUK cohort. The correlation with significance is listed for the coefficient (FDR-adjusted, *p* < 0.05). The correlation between the signature of PPS in the derivation cohort and total (poly)phenols from 7DDs is also listed for suggestive significance (FDR-adjusted, *p* = 0.06). PPS, (poly)phenol-rich dietary score; FFQ, food frequency questionnaire; and 7DD, 7-day food diary.

The correlation between (poly)phenol metabolic signatures and FFQ and 7DD estimated (poly)phenol intake in the derivation and validation data sets are shown in Figure 5. The (poly)phenol signatures and FFQ and 7DD estimated (poly)phenol intake were positively correlated, and the highest number of significant correlations were observed in the derivation data set. (Poly)phenol intake subclasses estimated from FFQs were all significantly linked with the metabolic signature in this data set 0.18 (0.06, 0.31) for lignans to 0.30 (0.18, 0.41) for phenolic acids, FDR-adjusted (*p* < 0.05), except for flavonoids [0.07 (−0.06, 0.19), FDR-adjusted *p* = 0.32]. For 7DDs, a trend for a significant correlation between total (poly)phenols and the metabolic signature was observed (FDR-adjusted *p* = 0.06). Lignans, other (poly)phenols, and stilbenes from 7DDs were positively linked with the signature [0.18 (0.03, 0.33) to 0.27 (0.12, 0.41)].

In the validation data set, the correlation between lignans from 7DDs and signature in the ABP cohort ranked highest [0.33 (0.14, 0.50), FDR-adjusted *p* < 0.01], followed by phenolic acids [0.30 (0.11, 0.48), FDR-adjusted *p* < 0.01], other (poly)phenols from FFQs [0.23 (0.03, 0.42), FDR-adjusted *p* = 0.03], and phenolic acids from 7DDs [0.22 (0.02, 0.40), FDR-adjusted *p* = 0.03]. Significant correlations were also found in the TwinsUK cohort, including other (poly)phenols, total (poly)phenols, phenolic acids, and stilbenes from FFQs with *r* ranging from 0.17 (0.03, 0.30) to 0.25 (0.11, 0.37) (FDR-adjusted *p* < 0.05). In the POLYNTAKE cohort with plasma samples, only flavonoids from FFQs were correlated with the metabolic signature [0.16 (0.02, 0.29), FDR-adjusted *p* = 0.02].

## Discussion

This is the first study to investigate and develop a metabolic signature to measure adherence to a (poly)phenol-rich diet based on targeted metabolomics data from multiple biofluids, including 24 h urine, plasma, and spot urine, in a UK population sample. The metabolic signature constituting 51 metabolites showed a stronger correlation with PPS than the individual biomarkers and was significantly correlated with PPS in the internal and external validation cohorts. The total (poly)phenol intake and each class of (poly)phenol intake assessed from FFQs or 7DDs were positively linked with metabolic signatures from at least one derivation or validation cohort. This result indicated the potential application of an array of metabolites as composite markers to assess adherence to a habitual (poly)phenol-rich diet in free-living populations.

More than 8000 different types of plant (poly)phenols have been identified,^31^ which increases the complexity of estimating dietary (poly)phenol intake. The PPS dietary pattern was developed to assess adherence to habitual (poly)phenol-rich dietary consumption with a comprehensive list of predefined (poly)phenol-rich food groups, i.e., tea, coffee, red wine, wholegrains, chocolate and cocoa products, berries, grapes, plums, citrus fruits, green vegetables, soy and products, nuts, and olive oil.^12^ Coherently, the 51 metabolites from 5 major (poly)phenol classes were identified as positively associated with PPS, thus assembling a representative set of metabolites to establish a (poly)phenol-rich diet signature.

As the predominant component of the PPS metabolic signature, phenolic acids accounted for most metabolites associated with PPS, including 18 hydroxycinnamic acids, 15 hydroxybenzoic acids, and six phenylacetic acids. Phenolic acids are abundant in various dietary sources, including coffee, tea, red wine, vegetables, and fruits.^32^ In the EPIC study, phenolic acids were the main contributors to total (poly)phenol intake in non-Mediterranean (MED) countries, representing 57 and 53% of the total among males and females, respectively.^33^ As the most consumed (poly)phenols among all age groups in the UK National Diet and Nutrition Survey (NDNS),^34^ hydroxycinnamic acids were also identified as having the highest number of associations with PPS among all subclasses in the present study, including 4-*O*-caffeoylquinic acid (cryptochlorogenic acid, abundant in coffee^35^), 4′-hydroxy-3′,5′-dimethoxycinnamic acid (sinapic acid, i.e., oranges, grapefruits, cranberries, and herbs^36^), 3′,4′-dihydroxycinnamic acids and derivatives (caffeic acids and derivatives, i.e., tea, wine, and coffee^37^), 4′/2′-hydroxycinnamic acid and derivatives (p/*o*-coumaric acid and derivatives, i.e., crops), and 3′/4′-methoxycinnamic acid and derivatives (iso/ferulic acid and derivatives, i.e., bread, fruits and cereals^33^). Hydroxybenzoic acids are commonly present in glycosylated forms, with tea as the predominant food source in the UK NDNS,^34^ compared to coffee as the major source of hydroxycinnamic acids among European regions.^33^ In our previous work with the same cohort (POLYNTAKE),^12^ the (poly)phenol-rich food items included in the PPS contributed 99.7% to total (poly)phenol intake, with tea and coffee contributing the most (33.7 and 44.2% of the total (poly)phenol, respectively). Due to the high contribution of tea and coffee, hydroxybenzoic and hydroxycinnamic acids accounted for the highest number of metabolites that were positively associated with PPS in this study. Moreover, phenolic acids are also gut microbial metabolites of nearly all (poly)phenol classes,^38^ which may also contribute to the strong association with PPS observed here.

Flavonoids represent one of the most diverse (poly)phenol groups, which cover more than half of the known (poly)phenol compounds^31^ and are commonly found in the UK diet, including flavanones (i.e., citrus fruit and juice), flavonols (i.e., tea, apples, and onions),^1^ and dihydrochalcones (i.e., apple and apple juice).^39^ These food groups were also included in the PPS due to their abundant (poly)phenol content. Here, several metabolites from these subclasses of flavonoids were positively linked with PPS, including naringenin-4′-glucuronide (flavanones), quercetin, quercetin-3-glucuronide, quercetin-7-glucuronide (flavonols), and phloretin (dihydrochalcones), indicating their potential to be biomarkers of a (poly)phenol-rich diet. Other abundant metabolites of flavonoids, e.g., epicatechin-3′-sulfate (one of the main metabolites of flavan-3-ols) and (4*R*)-5-(3′,4′-dihydroxyphenyl)-γ-valerolactone-4′-sulfate (proposed biomarker of flavan-3-ol intake^40^), were commonly found in tea drinkers in the UK population;^1^ however, they were not significantly associated with PPS. The discrepancies might be related to the population of this study, which had a relatively low tea consumption in this cohort (1.5 ± 1.6 cups per day), which might weaken the association between PPS and flavan-3-ol metabolites.

Other classes, including urinary metabolites classified as lignans, stilbenes, and other (poly)phenols with highly diverse chemical structures, were also positively associated with PPS. Lignans are a large group of compounds with two phenylpropene units coupled by a β–β′ bond, and the major food sources depend on the region, such as cereal products (24%) in non-Mediterranean countries and seeds (49%) in the UK population.^33^ In the human gut, lignans are metabolized into estrogenic enterolignans, enterodiol, and enterolactone (enterolactone-glucuronide, enterolactone-sulfate).^41^ These compounds are also widely distributed in plant-based foods, such as seeds, vegetables, fruits, legumes, and wholegrains.^42^ Thus, urinary enterolactone has been identified as a potential biomarker of plant-rich diet patterns in previous studies, such as MED, DASH, and AHEI-2010.^10,43^ Stilbenes possess a 1,4-diarylbutane structure. The most well-known stilbene is resveratrol, which has both *trans*- and *cis*-forms that appear naturally in food, with wine (i.e., *cis*-resveratrol-4′-glucuronide, dihydroresveratrol) as the prominent source (>92%) in all European regions.^33^ Other (poly)phenols have less typical chemical structures and include tyrosols, benzene diols and triols, benzaldehydes, and hydroxycoumarins in our research. These compounds could originate from very specific sources, e.g., tyrosols, or be generated by the gut-microbiome from multiple different classes of ingested (poly)phenols, e.g., ellagitannins, flavonoids, and phenolic acids during ring fission and cleavage of parent compounds.^44^ In the present study, these representative metabolites were positively associated with PPS, including enterodiol, enterolactone, enterolactone-glucuronide, enterolactone-sulfate (i.e., flaxseed and wholegrains), cis-resveratrol-4′-glucuronide, dihydroresveratrol (i.e., red wine), 2-(4-hydroxyphenyl)ethanol (tyrosols, i.e., olive oil), and 2-hydroxybenzene-1-glucuronide (i.e., coffee).

Metabolomics profiling, as a validated and high-throughput analytical methodology,^8^ was employed in this study to evaluate more than 100 food and microbiota-derived metabolites, including a large number of phase II metabolites, in biofluid 24 h urine, fasting plasma, and spot urine, which provided a snapshot of the (poly)phenol metabolome in a free-living UK population. To date, evidence has found a positive relationship between some of the metabolites discussed above, in particular hydroxybenzoic, hydroxycinnamic, phenylacetic, and hippuric acids and several plant-rich dietary patterns, such as Alternative HEI-2010 (AHEI-2010),^10^ DASH,^45^ PDI,^45^ MIND,^45^ and MDS.^45^ This overlap between PPS and other dietary patterns may be due to the shared plant-based food groups, such as fruits and vegetables, which highlight the importance of establishing multimetabolite panels of (poly)phenol biomarkers with a comprehensive metabolite profile across each class to be able to distinguish (poly)phenol-specific patterns from other plant-based diets.

The total (poly)phenol and each class of (poly)phenol intake estimated from FFQs and 7DDs in the derivation and three validation cohorts were further employed to test the application of the signature and presented positive correlations with the signature from at least one cohort, indicating that the PPS signature can serve as an assessment tool for (poly)phenol-rich diets. The plasma signature was different from the urine one, with very few metabolites correlating with PPS, which may be largely due to the diverse nature of these biofluids. Kinetic studies indicate that fasting plasma can only capture long half-life metabolites from common food sources,^46^ and compounds like epicatechin-3′-sulfate with short half-lives require constant ingestion to maintain a high concentration in plasma for detection.^47^ On the contrary, urine samples, in particular 24 h urine collection, can capture most compounds excreted from (poly)phenol-rich food sources within several days postconsumption.^48^ In line with this, the 24 h urine sample metabolites exhibited more associations with total (poly)phenols estimated from FFQs and 7DD. More correlations were found in the signature from the 24 h and spot urine samples, which may be attributed to the similar biofluid nature of the derivation cohort. Since the primary signature was derived from urine samples, more correlations were identified between (poly)phenol intake and the signature from the urine sample. Compared with 24 h urine, spot urine is easier to collect and thus is widely applied in multiple nutritional epidemiological studies. Due to the variation in the time difference between sample collection and (poly)phenol consumption, metabolite estimation is still challenging in free-living people. The capture of limited urinary excretion would restrict the application of spot urine, whereas 24 h urine would be a sufficient sample to reflect the most recent (poly)phenol consumption. Coherently, lignan intake estimated from FFQs only showed a significant correlation with 24 h urine samples in the POLYNTAKE cohort, but not in spot urine samples from the TwinsUK cohort. Furthermore, the diverse biofluid nature may also contribute to the coefficient of correlation between PPS and metabolic signature decline from the subcohort ABP (24 h urine), the TwinsUK cohort (spot urine), to the POLYNTAKE cohort (plasma), indicating the 24 h urine sample was the most suitable biofluid to reflect (poly)phenol consumption. The discrepancies may also partially be attributed to the difference in population, e.g., the TwinsUK cohort consists of middle-aged twin females.^17^

When comparing the metabolic signature with commonly used dietary assessment methods in observational studies, we found a larger number of correlations with (poly)phenol intake estimated from FFQs than 7DDs. Stronger correlations with total (poly)phenol estimated from FFQs are shown compared to 7DDs. As for the class of (poly)phenols, flavonoids, other (poly)phenols, and stilbenes estimated from FFQs but not from 7DDs were significantly linked with the signature from the POLYNTAKE cohort (plasma), subcohort ABP (24 h urine), and POLYNTAKE cohort (24 h urine), respectively. Based on the large proportion (39 out of 51) of the selected individual (poly)phenols from the phenolic acid class, the most robust correlation was observed between the urinary metabolic signature from all cohorts and phenolic acid intake, with a stronger correlation with phenolic acid intake estimated from FFQs than 7DDs. In our previous study comparing the agreement between FFQs and 7DDs,^21^ only total (poly)phenol and phenolic acid intake showed moderate agreement between tools, whereas poor agreement was exhibited among the rest of the classes, which is in line with the robust relationship between total (poly)phenol, phenolic acids, and the signature across cohorts with urine samples (24 h urine and spot urine). In addition, since the signature was established based on the PPS from FFQs, the same assessment tool might partially contribute to the stronger correlation with (poly)phenol intake estimated from FFQs. The EPIC FFQ collects habitual intake frequencies of a predefined food list but is prone to recall bias.^5^ The estimated food records by diaries require participants to provide detailed intake and portion sizes at the time of consumption, which eliminates recall bias but increases burdens on participants and researchers, including the high compliance to the protocol and the intensive work of converting food records, respectively.^49^ In previous EPIC study publications, the total (poly)phenol intake in the UK was estimated to be 1521 mg/day in healthy adults and around 1700 mg/day in the general population aged 35–74 years.^33^ Consistently, the total (poly)phenol intake assessed by FFQs and 7DDs in the present study ranged from 1620.2 ± 928.3 mg/day in men (FFQs) to 1750.1 ± 942.8 mg/day in women (7DDs). In addition, high positive correlations were found among the total and each class of (poly)phenols from FFQs and 7DDs, indicating an agreement between the tools.

This study is the first to develop a metabolic signature to evaluate adherence to a (poly)phenol-rich diet by differentiating participants into high- and low-intake levels using a large-scale metabolomics platform and dual dietary measurement tools (FFQs and 7DDs). The metabolomics profiling assembled by an array of (poly)phenol metabolites from urine/plasma samples enabled us to capture the accurate (poly)phenol-rich food-related metabolome objectively.^13^ The signatures deriving from machine-learning models with multiple biofluids may allow a further application to assess the metabolic response and adherence to the (poly)phenol-rich diet in randomized controlled trials and prospective studies in other countries.

We acknowledge that quantifying 51 metabolites may not be very practical for routine research, in particular, in large epidemiological studies, and it would be more feasible to implement a simpler signature containing fewer metabolites. Although a few metabolites may not represent the wide range of (poly)phenol subclasses present in the diet, based on the strength of the associations and wide representation from different subclasses, a simplified signature may include cinnamic acid-4′-sulfate (*p*-coumaric acid-4′-sulfate), enterolactone-glucuronide, dihydroresveratrol, 2-(4-hydroxyphenyl)ethanol (tyrosol), phloretin, 2-hydroxybenzene-1-glucuronide (catechol-1-glucuronide), and quercetin. Further work is needed to determine whether this shorter panel of metabolites is a good signature of (poly) phenol-rich diets.

This study has several limitations. First, due to the vast diversity of thousands of (poly)phenol compounds present in the diet, increasing the number of compounds in the current targeted metabolomic profile may lead to a more accurate capture of habitual (poly)phenol consumption. Second, the *a priori* PPS dietary pattern shares several food items with other plant-rich dietary patterns, which may restrict the specificity of the signature in the evaluation of a (poly)phenol-rich diet. The selected 51 metabolites in the metabolic signature are only a fraction of the thousands of plant (poly)phenols identified so far and are likely predominant for people eating in the UK since the PPS was established based on the UK diet, which may restrict its further application to other countries. Third, the assessment of (poly)phenol intake using FFQs has many limitations, such as self-reported recall bias. In terms of (poly)phenol metabolite assessment, a limitation was that we did not monitor 24 h urine collection compliance. Further research needs to be conducted in the general UK population group with diverse dietary intakes. Interventional feeding studies are needed to validate this signature with actual (poly)phenol consumption from a (poly)phenol-rich diet. Future replication in more countries with larger study populations is also warranted.

To conclude, this study showed that a metabolic signature derived from targeted metabolomics in urine and plasma samples in combination with dietary assessment methods has the potential to reflect adherence to (poly)phenol-rich diets.

## Data Availability

The TwinsUK cohort data used in this study were obtained by the Department of Twin Research at King’s College London. The data can be released to bona fide researchers using our normal procedures overseen by the Wellcome Trust and its guidelines as part of our core funding (https://twinsuk.ac.uk/resources-for-researchers/access-our-data/).

## Supporting Information Available

Detailed information of all contributing research studies (Table S1); characteristics of the ABP and TwinsUK cohort population (Table S2); comparison of correlation with PPS between metabolic signature and significant individual metabolites (Table S3); correlation between PPS, FFQ, and 7DD estimated (poly)phenol intake (Figure S1); association between the class of (poly)phenol and urinary metabolites (Figure S2) (PDF)

## References

[ref1] Williamson G. The role of polyphenols in modern nutrition. Nutr. Bull. 2017, 42 (3), 226–235. 10.1111/nbu.12278. 28983192 PMC5601283

[ref2] Grosso G.; Micek A.; Godos J.; Pajak A.; Sciacca S.; Galvano F.; Giovannucci E. L. Dietary Flavonoid and Lignan Intake and Mortality in Prospective Cohort Studies: Systematic Review and Dose-Response Meta-Analysis. Am. J. Epidemiol. 2017, 185 (12), 1304–1316. 10.1093/aje/kww207. 28472215

[ref3] Mompeo O.; Spector T. D.; Matey Hernandez M.; Le Roy C.; Istas G.; Le Sayec M.; Mangino M.; Jennings A.; Rodriguez-Mateos A.; Valdes A. M.; Menni C. Consumption of Stilbenes and Flavonoids is Linked to Reduced Risk of Obesity Independently of Fiber Intake. Nutrients 2020, 12 (6), 1871 10.3390/nu12061871. 32585900 PMC7353284

[ref4] Caruso G.; Godos J.; Privitera A.; Lanza G.; Castellano S.; Chillemi A.; Bruni O.; Ferri R.; Caraci F.; Grosso G. Phenolic Acids and Prevention of Cognitive Decline: Polyphenols with a Neuroprotective Role in Cognitive Disorders and Alzheimer’s Disease. Nutrients 2022, 14 (4), 819 10.3390/nu14040819. 35215469 PMC8875888

[ref5] Xu Y.; Le Sayec M.; Roberts C.; Hein S.; Rodriguez-Mateos A.; Gibson R. Dietary Assessment Methods to Estimate (Poly)phenol Intake in Epidemiological Studies: A Systematic Review. Adv. Nutr. 2021, 12 (5), 1781–1801. 10.1093/advances/nmab017. 33684195 PMC8483972

[ref6] Brennan L.; Hu F. B. Metabolomics-Based Dietary Biomarkers in Nutritional Epidemiology-Current Status and Future Opportunities. Mol. Nutr. Food Res. 2019, 63 (1), e1701064 10.1002/mnfr.201701064. 29688616

[ref7] Li J.; Guasch-Ferre M.; Chung W.; Ruiz-Canela M.; Toledo E.; Corella D.; Bhupathiraju S. N.; Tobias D. K.; Tabung F. K.; Hu J.; Zhao T.; Turman C.; Feng Y. A.; Clish C. B.; Mucci L.; Eliassen A. H.; Costenbader K. H.; Karlson E. W.; Wolpin B. M.; Ascherio A.; Rimm E. B.; Manson J. E.; Qi L.; Martinez-Gonzalez M. A.; Salas-Salvado J.; Hu F. B.; Liang L. The Mediterranean diet, plasma metabolome, and cardiovascular disease risk. Eur. Heart J. 2020, 41 (28), 2645–2656. 10.1093/eurheartj/ehaa209. 32406924 PMC7377580

[ref8] Guasch-Ferré M.; Bhupathiraju S. N.; Hu F. B. Use of Metabolomics in Improving Assessment of Dietary Intake. Clin. Chem. 2018, 64 (1), 82–98. 10.1373/clinchem.2017.272344. 29038146 PMC5975233

[ref9] Tong T. Y. N.; Koulman A.; Griffin J. L.; Wareham N. J.; Forouhi N. G.; Imamura F. A Combination of Metabolites Predicts Adherence to the Mediterranean Diet Pattern and Its Associations with Insulin Sensitivity and Lipid Homeostasis in the General Population: The Fenland Study, United Kingdom. J. Nutr. 2020, 150 (3), 568–578. 10.1093/jn/nxz263. 31665391 PMC7315099

[ref10] Castellano-Escuder P.; Gonzalez-Dominguez R.; Vaillant M. F.; Casas-Agustench P.; Hidalgo-Liberona N.; Estanyol-Torres N.; Wilson T.; Beckmann M.; Lloyd A. J.; Oberli M.; Moinard C.; Pison C.; Borel J. C.; Joyeux-Faure M.; Sicard M.; Artemova S.; Terrisse H.; Dancer P.; Draper J.; Sanchez-Pla A.; Andres-Lacueva C. Assessing Adherence to Healthy Dietary Habits Through the Urinary Food Metabolome: Results From a European Two-Center Study. Front. Nutr. 2022, 9, 880770 10.3389/fnut.2022.880770. 35757242 PMC9219016

[ref11] Noh H.; Freisling H.; Assi N.; Zamora-Ros R.; Achaintre D.; Affret A.; Mancini F.; Boutron-Ruault M. C.; Flogel A.; Boeing H.; Kuhn T.; Schubel R.; Trichopoulou A.; Naska A.; Kritikou M.; Palli D.; Pala V.; Tumino R.; Ricceri F.; Santucci de Magistris M.; Cross A.; Slimani N.; Scalbert A.; Ferrari P. Identification of Urinary Polyphenol Metabolite Patterns Associated with Polyphenol-Rich Food Intake in Adults from Four European Countries. Nutrients 2017, 9 (8), 796 10.3390/nu9080796. 28757581 PMC5579590

[ref12] Xu Y.; Li Y.; Hu J.; Gibson R.; Rodriguez-Mateos A. Development of a novel (poly)phenol-rich diet score and its association with urinary (poly)phenol metabolites. Food Funct. 2023, 14, 9635–9649. 10.1039/D3FO01982A. 37840467

[ref13] Domínguez-Fernández M.; Xu Y.; Young Tie Yang P.; Alotaibi W.; Gibson R.; Hall W. L.; Barron L.; Ludwig I. A.; Cid C.; Rodriguez-Mateos A. Quantitative Assessment of Dietary (Poly)phenol Intake: A High-Throughput Targeted Metabolomics Method for Blood and Urine Samples. J. Agric. Food Chem. 2021, 69 (1), 537–554. 10.1021/acs.jafc.0c07055. 33372779

[ref14] Li Y.; Xu Y.; Ma X.; Le Sayec M.; Wu H.; Dazzan P.; Nosarti C.; Heiss C.; Gibson R.; Rodriguez-Mateos A. (Poly)phenol intake, plant-rich dietary patterns and cardiometabolic health: a cross-sectional study. Food Funct. 2023, 14, 4078–4091. 10.1039/D3FO00019B. 37097300

[ref15] Banna J. C.; McCrory M. A.; Fialkowski M. K.; Boushey C. Examining Plausibility of Self-Reported Energy Intake Data: Considerations for Method Selection. Front. Nutr. 2017, 4, 45 10.3389/fnut.2017.00045. 28993807 PMC5622407

[ref16] Oliveira P. S.; Levy J.; Carli E.; Bensenor I. J. M.; Lotufo P. A.; Pereira R. A.; Yokoo E. M.; Sichieri R.; Crispim S. P.; Marchioni D. M. L. Estimation of underreporting of energy intake using different methods in a subsample of the ELSA-Brasil study. Cad. Saude Publica 2022, 38 (7), e00249821 10.1590/0102-311xen249821. 35894363

[ref17] Li Y.; Xu Y.; Le Roy C.; Hu J.; Steves C. J.; Bell J. T.; Spector T. D.; Gibson R.; Menni C.; Rodriguez-Mateos A. Interplay between the (Poly)phenol Metabolome, Gut Microbiome, and Cardiovascular Health in Women: A Cross-Sectional Study from the TwinsUK Cohort. Nutrients 2023, 15 (8), 1900 10.3390/nu15081900. 37111123 PMC10141398

[ref18] Bingham S. A.; Welch A. A.; McTaggart A.; Mulligan A. A.; Runswick S. A.; Luben R.; Oakes S.; Khaw K. T.; Wareham N.; Day N. E. Nutritional methods in the European Prospective Investigation of Cancer in Norfolk. Public Health Nutr. 2001, 4 (3), 847–858. 10.1079/PHN2000102. 11415493

[ref19] McKeown N. M.; Day N. E.; Welch A. A.; Runswick S. A.; Luben R. N.; Mulligan A. A.; McTaggart A.; Bingham S. A. Use of biological markers to validate self-reported dietary intake in a random sample of the European Prospective Investigation into Cancer United Kingdom Norfolk cohort. Am. J. Clin. Nutr. 2001, 74 (2), 188–196. 10.1093/ajcn/74.2.188. 11470719

[ref20] McCance R. A.; Widdowson E. M. McCance and Widdowson’s: The Composition of Foods, 6th ed.; Royal Society of Chemistry: Cambridge, 2002.

[ref21] Xu Y.; Li Y.; Ma X.; Alotaibi W.; Le Sayec M.; Cheok A.; Wood E.; Hein S.; Young Tie Yang P.; Hall W. L.; Nosarti C.; Dazzan P.; Gibson R.; Rodriguez-Mateos A. Comparison between dietary assessment methods and biomarkers in estimating dietary (poly)phenol intake. Food Funct. 2023, 14 (3), 1369–1386. 10.1039/D2FO02755K. 36655801

[ref22] Rothwell J. A.; Perez-Jimenez J.; Neveu V.; Medina-Remon A.; M’Hiri N.; Garcia-Lobato P.; Manach C.; Knox C.; Eisner R.; Wishart D. S.; Scalbert A. Phenol-Explorer 3.0: a major update of the Phenol-Explorer database to incorporate data on the effects of food processing on polyphenol content. Database 2013, 2013, bat070 10.1093/database/bat070. 24103452 PMC3792339

[ref23] Bhagwat S.; Haytowitz D. B. USDA Database for the Isoflavone Content of Selected Foods, release 2.1; Beltsville Human Nutrition Research Center, ARS, USDA, 2015.

[ref24] Bhagwat S.; Haytowitz D. B. USDA Database for the Proanthocyanidin Content of Selected Foods, release 2; Beltsville Human Nutrition Research Center, ARS, USDA, 2015.

[ref25] Bhagwat S.; Haytowitz D. B. USDA Database for the Flavonoid Content of Selected Foods, release 3.2; Beltsville Human Nutrition Research Center, ARS, USDA, 2016.

[ref26] R Core Team. R: A Language and Environment for Statistical Computing; R Core Team, 2013.

[ref27] Johnson W. E.; Li C.; Rabinovic A. Adjusting batch effects in microarray expression data using empirical Bayes methods. Biostatistics 2007, 8 (1), 118–127. 10.1093/biostatistics/kxj037. 16632515

[ref28] Han W.; Li L. Evaluating and minimizing batch effects in metabolomics. Mass Spectrom. Rev. 2022, 41 (3), 421–442. 10.1002/mas.21672. 33238061

[ref29] Li J.; Ji L. Adjusting multiple testing in multilocus analyses using the eigenvalues of a correlation matrix. Heredity 2005, 95 (3), 221–227. 10.1038/sj.hdy.6800717. 16077740

[ref30] Li C.; Imamura F.; Wedekind R.; Stewart I. D.; Pietzner M.; Wheeler E.; Forouhi N. G.; Langenberg C.; Scalbert A.; Wareham N. J. Development and validation of a metabolite score for red meat intake: an observational cohort study and randomized controlled dietary intervention. Am. J. Clin. Nutr. 2022, 116 (2), 511–522. 10.1093/ajcn/nqac094. 35754192 PMC9348983

[ref31] Cheynier V. Polyphenols in foods are more complex than often thought. Am. J. Clin. Nutr. 2005, 81 (1), 223S–229S. 10.1093/ajcn/81.1.223S. 15640485

[ref32] Zamora-Ros R.; Rothwell J. A.; Scalbert A.; Knaze V.; Romieu I.; Slimani N.; Fagherazzi G.; Perquier F.; Touillaud M.; Molina-Montes E.; Huerta J. M.; Barricarte A.; Amiano P.; Menendez V.; Tumino R.; de Magistris M. S.; Palli D.; Ricceri F.; Sieri S.; Crowe F. L.; Khaw K. T.; Wareham N. J.; Grote V.; Li K. R.; Boeing H.; Forster J.; Trichopoulou A.; Benetou V.; Tsiotas K.; Bueno-de-Mesquita H. B.; Ros M.; Peeters P. H. M.; Tjonneland A.; Halkjaer J.; Overvad K.; Ericson U.; Wallstrom P.; Johansson I.; Landberg R.; Weiderpass E.; Engeset D.; Skeie G.; Wark P.; Riboli E.; Gonzalez C. A. Dietary intakes and food sources of phenolic acids in the European Prospective Investigation into Cancer and Nutrition (EPIC) study. Br. J. Nutr. 2013, 110 (8), 1500–1511. 10.1017/S0007114513000688. 23507418

[ref33] Zamora-Ros R.; Knaze V.; Rothwell J. A.; Hemon B.; Moskal A.; Overvad K.; Tjonneland A.; Kyro C.; Fagherazzi G.; Boutron-Ruault M. C.; Touillaud M.; Katzke V.; Kuhn T.; Boeing H.; Forster J.; Trichopoulou A.; Valanou E.; Peppa E.; Palli D.; Agnoli C.; Ricceri F.; Tumino R.; de Magistris M. S.; Peeters P. H.; Bueno-de-Mesquita H. B.; Engeset D.; Skeie G.; Hjartaker A.; Menendez V.; Agudo A.; Molina-Montes E.; Huerta J. M.; Barricarte A.; Amiano P.; Sonestedt E.; Nilsson L. M.; Landberg R.; Key T. J.; Khaw K. T.; Wareham N. J.; Lu Y.; Slimani N.; Romieu I.; Riboli E.; Scalbert A. Dietary polyphenol intake in Europe: the European Prospective Investigation into Cancer and Nutrition (EPIC) study. Eur. J. Nutr. 2016, 55 (4), 1359–1375. 10.1007/s00394-015-0950-x. 26081647 PMC6284790

[ref34] Ziauddeen N.; Rosi A.; Del Rio D.; Amoutzopoulos B.; Nicholson S.; Page P.; Scazzina F.; Brighenti F.; Ray S.; Mena P. Dietary intake of (poly)phenols in children and adults: cross-sectional analysis of UK National Diet and Nutrition Survey Rolling Programme (2008–2014). Eur. J. Nutr. 2019, 58 (8), 3183–3198. 10.1007/s00394-018-1862-3. 30448880

[ref35] Afnan; Saleem A.; Akhtar M. F.; Sharif A.; Akhtar B.; Siddique R.; Ashraf G. M.; Alghamdi B. S.; Alharthy S. A. Anticancer, Cardio-Protective and Anti-Inflammatory Potential of Natural-Sources-Derived Phenolic Acids. Molecules 2022, 27 (21), 7286 10.3390/molecules27217286. 36364110 PMC9656250

[ref36] Pandi A.; Kalappan V. M. Pharmacological and therapeutic applications of Sinapic acid-an updated review. Mol. Biol. Rep. 2021, 48 (4), 3733–3745. 10.1007/s11033-021-06367-0. 33988797

[ref37] Mirzaei S.; Gholami M.; Zabolian A.; Saleki H.; Farahani M. V.; Hamzehlou S.; Far F. B.; Sharifzadeh S. O.; Samarghandian S.; Khan H.; Aref A. R.; Ashrafizadeh M.; Zarrabi A.; Sethi G. Caffeic acid and its derivatives as potential modulators of oncogenic molecular pathways: New hope in the fight against cancer. Pharmacol. Res. 2021, 171, 105759 10.1016/j.phrs.2021.105759. 34245864

[ref38] Lavefve L.; Howard L. R.; Carbonero F. Berry polyphenols metabolism and impact on human gut microbiota and health. Food Funct. 2020, 11 (1), 45–65. 10.1039/C9FO01634A. 31808762

[ref39] Ulaszewska M.; Vazquez-Manjarrez N.; Garcia-Aloy M.; Llorach R.; Mattivi F.; Dragsted L. O.; Pratico G.; Manach C. Food intake biomarkers for apple, pear, and stone fruit. Genes Nutr. 2018, 13, 29 10.1186/s12263-018-0620-8. 30519365 PMC6267079

[ref40] Favari C.; Mena P.; Curti C.; Istas G.; Heiss C.; Del Rio D.; Rodriguez-Mateos A. Kinetic profile and urinary excretion of phenyl-gamma-valerolactones upon consumption of cranberry: a dose-response relationship. Food Funct. 2020, 11 (5), 3975–3985. 10.1039/D0FO00806K. 32396592

[ref41] Carreau C.; Flouriot G.; Bennetau-Pelissero C.; Potier M. Enterodiol and enterolactone, two major diet-derived polyphenol metabolites have different impact on ERalpha transcriptional activation in human breast cancer cells. J. Steroid Biochem. Mol. Biol. 2008, 110 (1–2), 176–185. 10.1016/j.jsbmb.2008.03.032. 18457947

[ref42] Rodríguez-García C.; Sanchez-Quesada C.; Toledo E.; Delgado-Rodriguez M.; Gaforio J. J. Naturally Lignan-Rich Foods: A Dietary Tool for Health Promotion?. Molecules 2019, 24 (5), 917 10.3390/molecules24050917. 30845651 PMC6429205

[ref43] Whitton C.; Ho J. C. Y.; Rebello S. A.; van Dam R. M. Relative validity and reproducibility of dietary quality scores from a short diet screener in a multi-ethnic Asian population. Public Health Nutr. 2018, 21 (15), 2735–2743. 10.1017/S1368980018001830. 30081973 PMC10260988

[ref44] Del Rio D.; Rodriguez-Mateos A.; Spencer J. P.; Tognolini M.; Borges G.; Crozier A. Dietary (poly)phenolics in human health: structures, bioavailability, and evidence of protective effects against chronic diseases. Antioxid. Redox Signaling 2013, 18 (14), 1818–1892. 10.1089/ars.2012.4581. PMC3619154 22794138

[ref45] Li Y.; Xu Y.; Ma X.; Le Sayec M.; Wu H.; Dazzan P.; Nosarti C.; Heiss C.; Gibson R.; Rodriguez-Mateos A. (Poly)phenol intake, plant-rich dietary patterns and cardiometabolic health: a cross-sectional study. Food Funct. 2023, 14 (9), 4078–4091. 10.1039/D3FO00019B. 37097300

[ref46] Manach C.; Williamson G.; Morand C.; Scalbert A.; Rémésy C. Bioavailability and bioefficacy of polyphenols in humans. I. Review of 97 bioavailability studies. Am. J. Clin. Nutr. 2005, 81 (1), 230S–242S. 10.1093/ajcn/81.1.230S. 15640486

[ref47] Manach C.; Scalbert A.; Morand C.; Rémésy C.; Jiménez L. Polyphenols: food sources and bioavailability. Am. J. Clin. Nutr. 2004, 79 (5), 727–747. 10.1093/ajcn/79.5.727. 15113710

[ref48] Shen J.; Shan J.; Zhong L.; Liang B.; Zhang D.; Li M.; Tang H. Dietary Phytochemicals that Can Extend Longevity by Regulation of Metabolism. Plant Foods Hum. Nutr. 2022, 77 (1), 12–19. 10.1007/s11130-021-00946-z. 35025006 PMC8756168

[ref49] Shim J. S.; Oh K.; Kim H. C. Dietary assessment methods in epidemiologic studies. Epidemiol. Health 2014, 36, e2014009 10.4178/epih/e2014009. 25078382 PMC4154347

