# Supplementary material for: Development of a (Poly)phenol Metabolic Signature for Assessing (Poly)phenol-Rich Dietary Patterns
Source: J Agric Food Chem. 2024 Jun 3;72(23):13439–50. doi: 10.1021/acs.jafc.4c00959 (PMC11181312; doi:10.1021/acs.jafc.4c00959)
Supplement: Supplementary file 1 — jf4c00959_si_001.pdf [file jf4c00959_si_001.pdf]

Supporting Information

Development of a (poly)phenol metabolic signature for assessing (poly)phenol-rich dietary patterns

Yong Li [1], Yifan Xu [1], Melanie Le Sayec [1], Xinyu Yan[2], Tim D Spector [2], Claire J Steves [2], Jordana T Bell [2], Kerrin S Small [2], Cristina Menni [2], Rachel Gibson [1], Ana Rodriguez-Mateos [1, *]

[1] Department of Nutritional Sciences, School of Life Course and Population Sciences, Faculty of Life Sciences and Medicine, King's College London, London, UK, SE1 9NH

yong.3.li@kcl.ac.uk (Y.L.); yifan.xu@kcl.ac.uk (Y.X.);

melanie.le_sayec@kcl.ac.uk (M.L.S.); rachel.gibson@kcl.ac.uk (R.G.)

[2] Department of Twin Research & Genetic Epidemiology, School of Life Course and Population Sciences, Faculty of Life Sciences and Medicine, King's College London, London, UK, SE1 7EH

xinyu.yan@kcl.ac.uk (X.Y.); tim.spector@kcl.ac.uk (T.D.S.); claire.j.steves@kcl.ac.uk (C.J.S.); jordana.bell@kcl.ac.uk (J.T.B.); kerrin.small@kcl.ac.uk (K.S.S.); cristina.menni@kcl.ac.uk (C.M.)

[*] Address correspondence to Ana Rodriguez-Mateos, PhD, Department of Nutritional Sciences, School of Life Course and Population Sciences, Faculty of Life Sciences and Medicine, King's College London, London, UK, SE1 9NH, E-mail: ana.rodriguez-mateos@kcl.ac.uk

Table of Contents

Table S1 Detailed information of all contributing research studies.

Table S2 Characteristics of the ABP and TwinsUK cohort population.

Table S3 Comparison of correlation with PPS between metabolic signature and significant individual metabolites.

Figure S1 Correlation between PPS, FFQ and 7DD estimated (poly)phenol intake.

Figure S2 Association between the class of (poly)phenol and urinary metabolites.

Table S1. Detailed information of all contributing research studies

Study details	POLYNTAKE Study	Aronia Blood Pressure (ABP) study	TwinsUK study
Study design	Cross-sectional study [a]	2-arm, double-blind, parallel randomized controlled trial [b]	Longitudinal study [c]
Sample size	229	95	200
Biosamples	24h urine and fasting plasma	Baseline 24h urine and fasting plasma (samples collected post-intervention were not included in the present work)	Spot urine
Intervention	--	Participants consumed 1 capsule of the interventional product (aronia berry extract) or control (maltodextrin) daily for 12 weeks.	--
Dietary assessment tool	EPIC-Norfolk FFQ and 7DD	EPIC-Norfolk FFQ and 7DD	EPIC-Norfolk FFQ

Note: EPIC-Norfolk FFQ, (European Prospective Investigation into Cancer)-Norfolk food frequency questionnaire; 7DD, 7-day food diary.

a. The POLYNTAKE study consists of the baseline data of a series of clinical studies conducted at KCL using the same methodology for plasma and urine (poly)phenol metabolite analysis and dietary assessment. The ABP study is included in the POLYNTAKE study. Here, we used the 24h urine samples from POLYNTAKE as the derivation dataset and the fasting plasma as the internal validation dataset.

b. Aronia Berry Consumption on Blood Pressure study (ABP) is a clinical study conducted to explore the effect of Aronia Berry on vascular health (blood pressure). In this research, we only used the baseline data of the ABP study, prior to intervention, as well as the EPIC-Norfolk FFQ and 7DD collected at baseline for estimation of (poly)phenol intake.

c. TwinsUK, also known as TwinsUK Registry, is the biggest UK adult registry of twins in the United Kingdom, ages 16 to 98, to study the genetic and environmental aetiology of age-related complex traits and diseases. We analysed (poly)phenol metabolites in 200 urine samples from the TwinsUK study and used the collected EPIC-Norfolk FFQ from the same volunteers for estimation of (poly)phenol intake using the same methodology as the POLYNTAKE and ABP studies.

Table S2. Characteristics of the ABP and TwinsUK cohort population PPS, (Poly)phenol-rich dietary score; FFQ, Food frequency questionnaire

Characteristics	ABP study	TwinsUK study
Age (years)	56.4 (8.9)	62.0 (9.9)
Gender (n)		
Male	46 (48.4)	--
Female	49 (51.6)	200
Ethnicity (n)		
White	76 (80.0)	198 (99.0)
Black	8 (8.4)	0
Asian	8 (8.4)	0
Mixed	3 (3.2)	2 (1.0)
Energy intake (kcal/d)	1681.3 (490.7)	1782.5 (544.3)
Biosample (n)		
24h urine	95	--
Spot urine	--	200
PPS and estimated total (poly)phenol		
PPS	54.6 (11.4)	50.7 (11.7)
Total (poly)phenol estimated from FFQs	1898.6 (490.7)	2128.9 (964.1)
Total (poly)phenol estimated from 7DDs	1799.7 (1062.2)	--

Table S3. Comparison of correlation with PPS between metabolic signature and significant individual metabolites

Class	Subclass	Individual metabolite (common name)	Individual metabolite (recommended name)	PPS
		**Metabolic signature**		0.328
Flavonoids	Dihydrochalcones	Phloretin	Phloretin	0.198
Flavonoids	Flavanones	Naringenin-4'-glucuronide	Naringenin-4'-glucuronide	0.148
Flavonoids	Flavonols	Quercetin-3-glucuronide	Quercetin 3-glucuronide	0.173
Flavonoids	Flavonols	Quercetin-7-glucuronide	Quercetin 7-glucuronide	0.162
Flavonoids	Flavonols	Quercetin	Quercetin	0.185
Lignans	Lignans	Enterodiol	Enterodiol	0.162
Lignans	Lignans	Enterolactone-glucuronide	Enterolactone-glucuronide	0.254
Lignans	Lignans	Enterolactone-sulfate	Enterolactone-sulfate	0.248
Other (poly)phenol	Benzene diols and triols	Catechol-1-glucuronide	2-Hydroxybenzene-1-glucuronide	0.238
Other (poly)phenol	Tyrosols	Tyrosol	2-(4-hydroxyphenyl)ethanol	0.240
Phenolic acids	Cinnamic acid	Cinnamic acid	Cinnamic acid	0.224
Phenolic acids	Cinnamic acid	Caffeic acid-4'-sulfate	3'-Hydroxycinnamic acid-4'-sulfate	0.177
Phenolic acids	Cinnamic acid	Caffeic acid-4'-glucuronide	3'-Hydroxycinnamic acid-4'-glucuronide	0.203
Phenolic acids	Cinnamic acid	Caffeic acid-3'-sulfate	4'-Hydroxycinnamic acid-3'-sulfate	0.148
Phenolic acids	Cinnamic acid	p-Coumaric acid-4'-sulfate	Cinnamic acid-4'-sulfate	0.323
Phenolic acids	Cinnamic acid	p-Coumaric acid-4'-glucuronide	Cinnamic acid-4'-glucuronide	0.277
Phenolic acids	Cinnamic acid	Caffeic acid-3'-glucuronide	4'-Hydroxycinnamic acid-3'-glucuronide	0.229
Phenolic acids	Cinnamic acid	Ferulic acid-4'-sulfate	3'-Methoxycinnamic acid-4'-sulfate	0.179
Phenolic acids	Cinnamic acid	trans-Ferulic acid	4'-Hydroxy-3'-methoxycinnamic acid	0.145
Phenolic acids	Cinnamic acid	Caffeic acid	3',4'-Dihydroxycinnamic acid	0.233
Phenolic acids	Cinnamic acid	Cryptochlorogenic acid	4-O-Caffeoylquinic acid	0.193
Phenolic acids	Cinnamic acid	Isoferulic acid-3'-sulfate	4'-Methoxycinnamic acid-3'-sulfate	0.156
Phenolic acids	Cinnamic acid	Isoferulic acid-3'-glucuronide	4'-Methoxycinnamic acid-3'-glucuronide	0.227
Phenolic acids	Cinnamic acid	p-Coumaric acid	4'-Hydroxycinnamic acid	0.159
Phenolic acids	Cinnamic acid	Ferulic acid-4'-glucuronide	3'-Methoxycinnamic acid-4'-glucuronide	0.157
Phenolic acids	Cinnamic acid	Isoferulic acid	3'-Hydroxy-4'-methoxycinnamic acid	0.184
Phenolic acids	Cinnamic acid	Sinapic acid	4'-Hydroxy-3',5'-dimethoxycinnamic acid	0.192
Phenolic acids	Cinnamic acid	o-Coumaric acid	2'-Hydroxycinnamic acid	0.317
Phenolic acids	Hippuric acid	Hippuric acid	Hippuric acid	0.246
Phenolic acids	Hippuric acid	2'-Hydroxyhippuric acid	2'-Hydroxyhippuric acid	0.150
Phenolic acids	Hydroxybenzoic acids	Protocatechuic acid-4-sulfate	3-Hydroxybenzoic acid-4-sulfate	0.220
Phenolic acids	Hydroxybenzoic acids	Protocatechuic acid-3-sulfate	4-Hydroxybenzoic acid-3-sulfate	0.202
Phenolic acids	Hydroxybenzoic acids	2,3-Dihydroxybenzoic acid	2,3-Dihydroxybenzoic acid	0.240
Phenolic acids	Hydroxybenzoic acids	Protocatechuic acid-3-glucuronide	4-Hydroxybenzoic acid-3-glucuronide	0.168
Phenolic acids	Hydroxybenzoic acids	2,5-Dihydroxybenzoic acid	2,5-Dihydroxybenzoic acid	0.217
Phenolic acids	Hydroxybenzoic acids	2-Hydroxybenzoic acid	2-Hydroxybenzoic acid	0.274
Phenolic acids	Hydroxybenzoic acids	Isovanillic acid-3-sulfate	4-Methoxybenzoic acid-3-sulfate	0.242
Phenolic acids	Hydroxybenzoic acids	2,6-Dihydroxybenzoic acid	2,6-Dihydroxybenzoic acid	0.206
Phenolic acids	Hydroxybenzoic acids	2,4-Dihydroxybenzoic acid	2,4-Dihydroxybenzoic acid	0.208
Phenolic acids	Hydroxybenzoic acids	Vanillic acid	4-Hydroxy-3-methoxybenzoic acid	0.191
Phenolic acids	Hydroxybenzoic acids	Syringic acid	4-Hydroxy-3,5-dimethoxybenzoic acid	0.196
Phenolic acids	Hydroxybenzoic acids	2-Hydroxy-4-methoxybenzoic acid	2-Hydroxy-4-methoxybenzoic acid	0.233
Phenolic acids	Hydroxybenzoic acids	2,3,4-Trihydroxybenzoic acid	2,3,4-Trihydroxybenzoic acid	-0.164
Phenolic acids	Phenylacetic acid	Dihydrocaffeic acid	3-(3',4'-Dihydroxyphenyl)propanoic acid	0.225
Phenolic acids	Phenylacetic acid	Dihydrocaffeic acid-3'-glucuronide	3-(4'-Hydroxyphenyl)propanoic acid-3'-glucuronide	0.167
Phenolic acids	Phenylacetic acid	2-(4'-Hydroxyphenoxy)propanoic acid	2-(4'-Hydroxyphenoxy)propanoic acid	0.206
Phenolic acids	Phenylacetic acid	3-(2',3'-Dihydroxyphenyl)propanoic acid	3-(2',3'-Dihydroxyphenyl)propanoic acid	0.188
Phenolic acids	Phenylacetic acid	Dihydrocaffeic acid-3'-sulfate	3-(4'-Hydroxyphenyl)propanoic acid-3'-sulfate	0.209
Phenolic acids	Phenylacetic acid	3-(3'-Hydroxyphenyl)propanoic acid	3-(3'-Hydroxyphenyl)propanoic acid	0.154
Stilbenes	Stilbenes	Dihydroresveratrol	Dihydroresveratrol	0.241
Stilbenes	Stilbenes	cis-Resveratrol-4'-glucuronide	cis-Resveratrol-4'-glucuronide	0.178

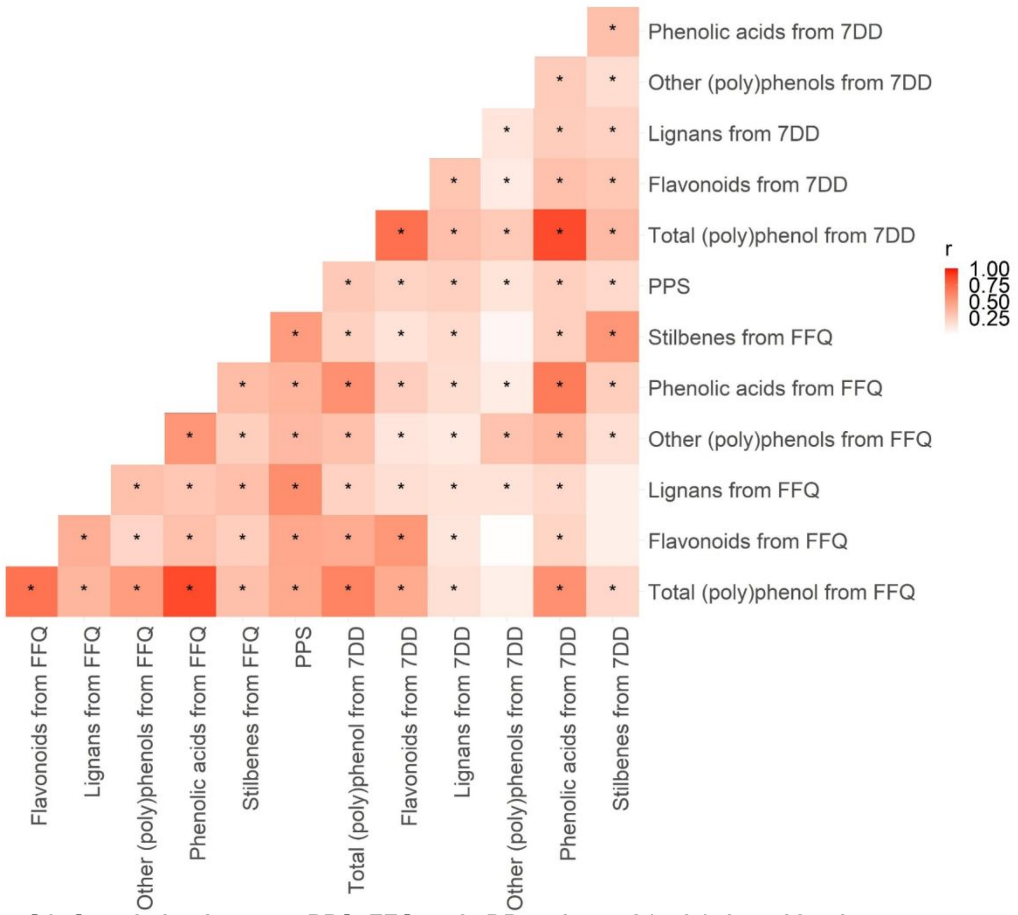

Figure S1. Correlation between PPS, FFQ and 7DD estimated (poly)phenol intake.

The colour scale indicates the Spearman correlation coefficient between FFQ and 7DD estimated total and class of (poly)phenol. Red colour illustrated respectively positive correlation and colour intensity represented the degree of the coefficient. The asterisks showed significance (*fdr-adjusted, $p < 0.05$), PPS, (Poly)phenol-rich dietary score; FFQ, Food frequency questionnaire; 7DD, 7-day food diary.

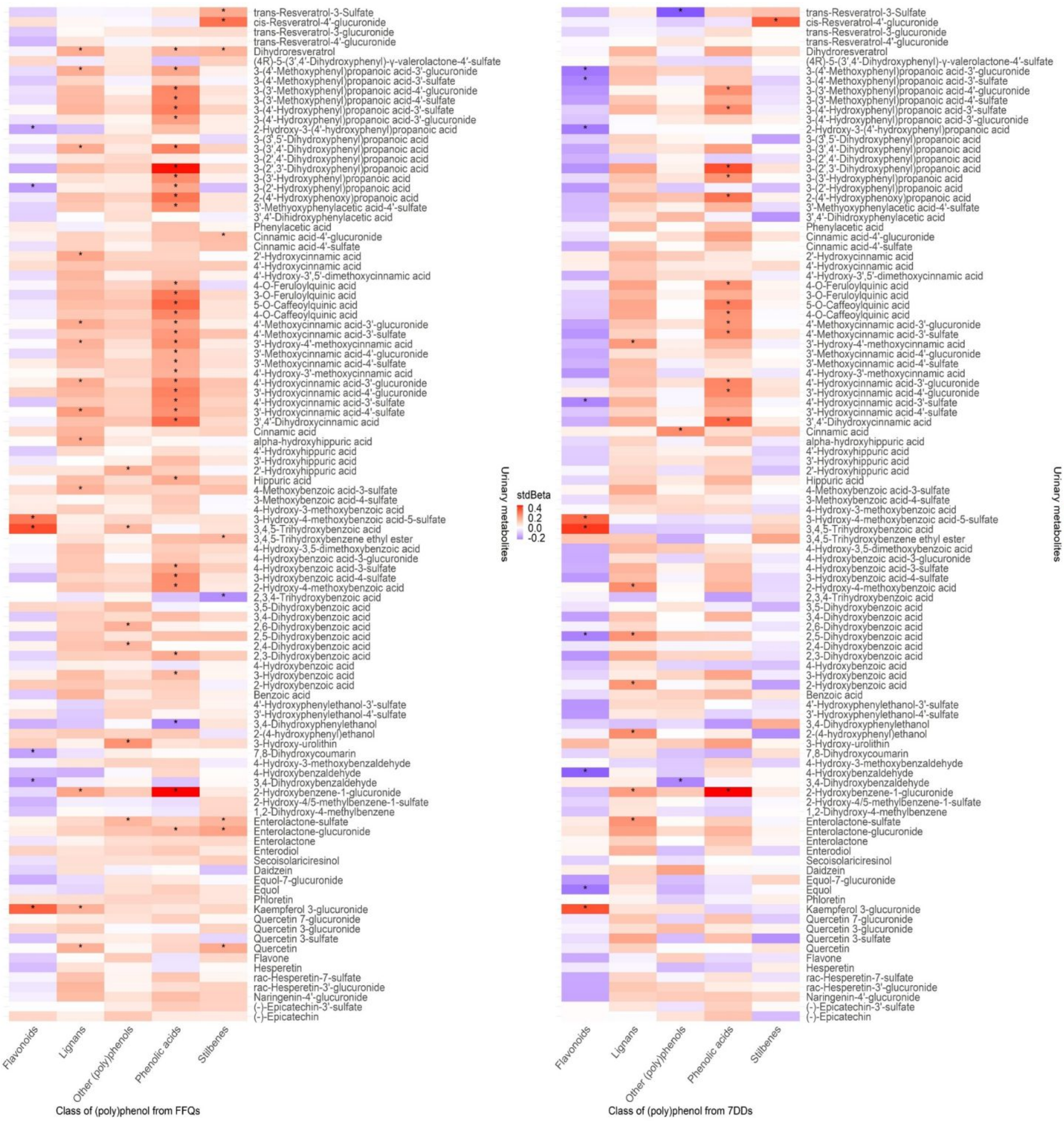

Figure S2. Association between the class of (poly)phenol and urinary metabolites.

The heatmap was plotted according to the standardized regression coefficients (stdBeta). The colour scale indicates the effect (stdBeta) of each urinary metabolite on the class of (poly)phenol. Red and blue illustrate positive and negative effects, and colour intensity represents the degree of effect. The asterisks showed significance (*: fdr-adjusted $p < 0.05$). FFQs, Food frequency questionnaires; 7DDs, 7-day food diaries. The associations were adjusted for energy intake.
